# Influence of Different Thermal Aging Conditions on Soot Combustion with Catalyst by Thermogravimetric Analysis

**DOI:** 10.3390/ma14133647

**Published:** 2021-06-30

**Authors:** Yi Yang, Jia Fang, Junfeng Huang, Zihan Qin, Qian Zhang, Ping Pu, Suozhu Pan

**Affiliations:** 1 Key Laboratory of Fluid and Power Machinery, Ministry of Education, School of Energy and Power Engineering, Xihua University, Chengdu 610039, China; yiyang11104@outlook.com (Y.Y.); SYX20040304150@outlook.com (J.H.); qwertyuiop123ty@outlook.com (Z.Q.); zltdkjmhcjly1@outlook.com (Q.Z.); asdfghjkl111112001@outlook.com (P.P.); suozhup@163.com (S.P.); 2 Vehicle Measurement, Control and Safety Key Laboratory of Sichuan Province, School of Automobile and Transportation, Xihua University, Chengdu 610039, China

**Keywords:** soot, diesel particulate filter, regeneration, Pt/Al_2_O_3_ catalyst, thermal aging, thermogravimetric analysis

## Abstract

Diesel particulates are deposited in the diesel particulate filter and removed by the regeneration process. The Printex-U (PU) particles are simulated as the diesel soot to investigate the influence of thermal aging conditions on soot combustion performance with the addition of catalysts. The comprehensive combustion index *S*, combustion stability index *R_w_* and peak temperature *T_p_* are obtained to evaluate the combustion performance. Compared with the PU/Pt mixtures of different Pt contents (2 g/ft^3^, 3.5 g/ft^3^, and 5 g/ft^3^), the 10 g/ft^3^ Pt contents improve soot combustion with the outstanding oxygen absorption ability. When the weight ratio of PU/Pt mixture is 1:1, the promoted effect achieves the maximum degree. The *S* and *R_w_* increase to 8.90 × 10^−8^ %^2^min^−2^°C^−3^ and 39.11 × 10^5^, respectively, compared with pure PU. After the thermal aging process, the PU/Pt mixture with a 350 °C aging temperature for 10 h promotes the soot combustion the best when compared to pure PU particles. It is not good as the PU/Pt mixture without aging, because the inner properties of soot and Pt/Al_2_O_3_ catalyst may have been changed. The *S* and *R_w_* are 9.07 × 10^−8^ %^2^min^−2^°C^−3^ and 38.39 × 10^5^, respectively, which are close to the no aging mixture. This work plays a crucial role in understanding the mechanism of the comprehensive effect of soot and catalyst on soot combustion after the thermal aging process.

## 1. Introduction

Diesel engines have found an increasingly wide utilization in the field of industry because of their high thermal efficiency and preferable engine performance [1]. However, the particulate matter (PM) emission of diesel engines is higher than that of gasoline engines [2]. The government has formulated a series of extremely stringent emission standards to direct against the emission problem. This problem caused by diesel engines is harmful to human health and the environment [3,4]. A wall-flow diesel particulate filter (DPF) can dispose of those emission problems efficiently [5]. However, the exhaust back pressure of DPF increases sharply, and the filtration performance decreases with the continuous deposition of PM [6], which leads to the decrease of engine thermal efficiency and odious emission performance [7,8]. Consequently, it is imperative to remove soot particles and reduce exhaust back pressure periodically by the regeneration of the DPF [9,10]. The active regeneration [11], passive regeneration and active/passive hybrid regeneration [12] are the three main regeneration methods to keep the filter in regular work. The regeneration process is not a transient process [13], and the exhaust temperature of the diesel engine is usually between 180 °C to 400 °C [14,15] so that the PM in DPF cannot be removed and oxidized immediately [16]. When PM is deposited in the channel of the DPF, the physical and chemical properties of complicated compositions will be transformed under the action of thermal aging temperature exhaust gas [17,18], which makes the oxidation reaction characteristics of deposited soot more complex [19]. In this study, the oxidation performance of soot/catalyst mixtures after thermal aging process are studied by thermogravimetric analysis and kinetic analysis.

The issue of removing soot particles deposited in the DPF with catalysts. For example, Pt/Al_2_O_3_ has received considerable critical attentions because of the superior catalytic activity and selectivity. Álvarez-Docio et al. [20] reports that Pt/Al_2_O_3_ catalyst is the most widely used catalyst for soot oxidation due to its high activity and durability. The soot combustion is related to the contract area among gaseous oxidants, soot and active sites. Moreover, the Pt catalysts are the most sulfur resistant soot combustion catalysts, which are capable of tolerating a large amount of SO_2_ [21,22]. However, the thermal aging effect of the DPF has been a largely under explored domain. Dacosta et al. [23] suggests the filtration efficiency, pressure drop, and regeneration efficiency of the DPF demonstrate little significant change through the thermal aging process at 550 °C or 650 °C for 100 h. Dimitrios et al. [24] implicates that the filtration and regeneration performance of catalytic diesel particulate filters (CDPF) with the cerium-based oxide catalyst decreased obviously after thermal aging. Thermal aging processes not only influence the performance of the DPF but also the physical and chemical properties of soot and catalysts deposited in the DPF. Soussi et al. [25] defines soot surface aging decreases of active sites or defect sites on soot particles surface. The thermal aging soot effect is related to the local temperature and the residence time. Surovikin et al. [26] reveals that the specific adsorption surface of nano-dispersed carbon black samples decreases to geometrical one after thermal treatment. Dacquin et al. [27] proposes that the small Pt particles can interact with supports strongly after thermal aging treatment, and these preferred orientations can protect them from sintering under high-temperature conditions. Wiebenga et al. [28] suggests that the size of Pt–Pd alloy particles will increase because they are far away from the inlet surface in the process of DPF regeneration. However, the above investigations are not enough to illustrate the effect of thermal aging treatment on soot/catalyst mixtures in DPF, and the combustion performance of PU/Pt mixtures in the N_2_/O_2_ atmosphere has not studied by the above studies.

All the above studies have largely been focused on soot oxidation and the influence of catalyst or thermal aging DPFs alone on soot combustion. Yet, the interaction effect of soot/catalyst mixtures with thermal aging is still unclear, which is equally inevitable towards the regeneration process. The novelty of this study is to investigate the effect of soot/catalyst mixtures’ aging process on soot combustion by thermogravimetric experiments in N_2_/O_2_ atmosphere. With the change of thermal aging temperatures and times, the combustion characteristics of soot/catalyst mixtures can be evaluated based on the TGA analyzer. The purpose of this study is to provide a theoretical basis for the thermal aging process of DPF regeneration, and it can provide some theoretical guidance for the improvement strategy of DPF catalyst.

## 2. Description of Experiments

### 2.1. Materials and Samples Preparation

The soot particles in DPFs are mainly composed of complex constituents such as the agglomerated particles, and it is hard to collect [29]. Printex-U (PU), as the classical commercial synthetic soot, is employed in this experiment, which is sourced from Degussa Gmbh company, (EVONIK, Shanghai, China). It is reported that the physical and chemical properties of PU particles are similar to the real soot particles from diesel engine [30] and the detailed parameters of PU are listed in Table 1.

The commercial catalysts (mainly composed of Al_2_O_3_, platinum) are sourced from Texas Emission Control Technology (Wuxi) Co., Ltd., (Texas Emission Solutions (Wuxi), Wuxi, China), with different contents of platinum. The Pt contents are 2 g/ft^3^, 3.5 g/ft^3^, 5 g/ft^3^ and 10 g/ft^3^, respectively. This kind of catalyst is sintered with slurry instead of being directly coated on the ceramic carrier. The main parameters of catalysts are shown in Table 2.

In order to remove the moisture and water vapor in the sample, all the PU/Pt samples are supposed to dry in a vacuum oven at 110 °C for 2 h. After the preparation, the samples are shaken in a vortex mixer three times, and each duration is approximately 10 min to obtain a superior consistency. The mixing method of PU/Pt mixtures is tight-contact mode by the above preparation steps, and this method ensures the sufficient contact between PU particles and Pt catalysts.

### 2.2. Apparatus

**Figure 1:**
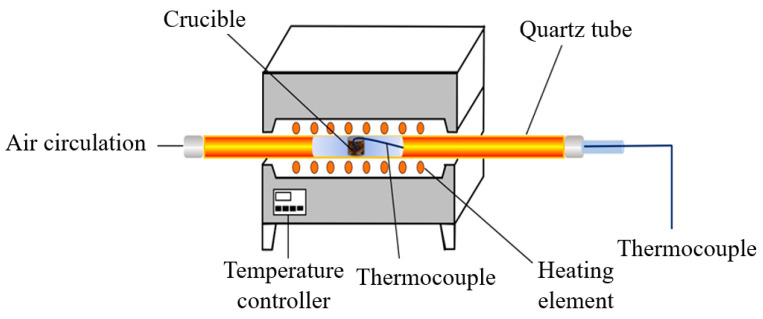
The fixed bed system diagram.

**Figure 2:**
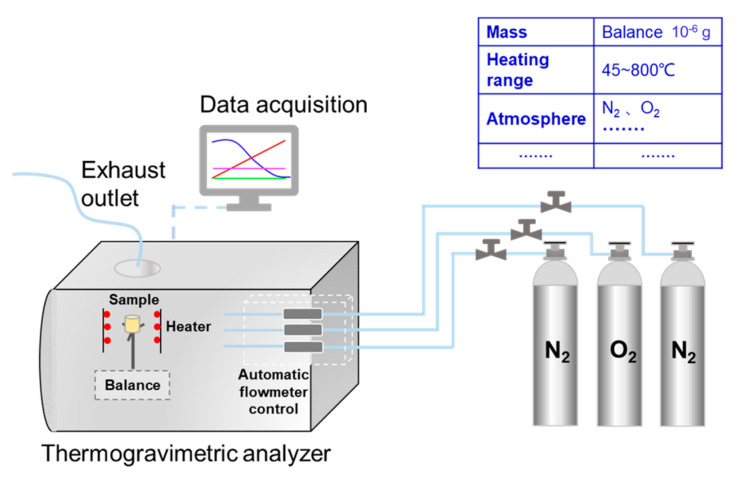
The schematic diagram of TG209F3.

The fixed bed system and TGA analyzer in this experiment are shown in Figure 1 and Figure 2, respectively. The fixed bed instrument is used to obtain the mixtures heated in the N_2_/O_2_ atmosphere for thermal aging, which can be heated to the desirable temperature and remain for a fixed time, set through judgement. The mixtures are put into a heat-resistant glass bottle and lean towards the inner edge of crucible in the fixed bed. This confirms the process of heating steadily and uniformly. The thermal aging experiment is carried out by the automatic heating system with controlled time. The set temperature of thermal aging process is 5 °C higher than the desirable temperature to offset the heat losses of the gas flow.

The TGA analyzer (TG209F3, NETZSCH, Braustuberl, Germany) is applied to analyze the combustion characteristics of the samples. The temperature precision of TG analyzer is ±0.1 °C and its microbalance sensitivity is less than 0.1 μg, which ensures the accuracy and reliability of the experimental data. The mass of PU particles is fixed at 3 mg in each test, and the samples are prepared carefully according to the proportion. Then, the mixture is placed in the alumina crucible (diameter × height: 6.8 mm × 7.4 mm) and heated from 45 to 800 °C at the 10 °C min^−1^ heating ramp. To ensure the stability of the instrument, the 10 min remaining time at the start and end of each experiment is carried out by computer program. The inlet flow rate, oxygen concentration and heating rate are 100 mL min^−1^, 10% and 10 °C min^−1^, respectively [31,32]. As the protective gas, the concentration of nitrogen is 90%.

### 2.3. Data Analysis

**Figure 3:**
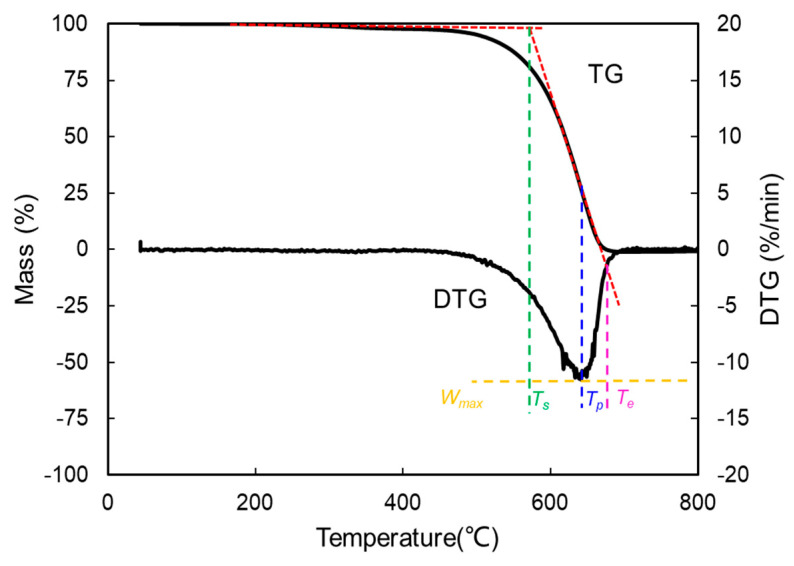
Definition of characteristic parameters in TG-DTG curves of soot oxidation.

With the increasing temperature, TG curves are achieved by recording the mass loss of the mixture continually, and the derivative thermogravimetric (DTG) curves are obtained by differentiating the TG curves. The starting temperature (*T_s_*), ending temperature (*T_e_*), and peak temperature (*T_p_*) are extracted to evaluate the oxidation performance [33] as identified in Figure 3.

*T_s_*, *T_e_* and *T_p_* represent the temperature when the mixture starts to burn, the temperature corresponding to the end of combustion process and the temperature of maximum weight loss, respectively. The comprehensive combustion index (*S*) can estimate the ignition, combustion and burnout properties, and the calculation is as follows [34]:

(1) $$S=\frac{W_{max}\times W_{mean}}{T_{s}^{2}\times T_{e}}$$

In this calculation, *W_max_*, *W_mean_*, *T_s_* and *T_e_* represent the maximum mass loss rate, the mean mass loss rate, the starting temperature and the ending temperature, respectively. The higher the values of *S*, the more preferable the combustion performance of the PU particles is.

The combustion stability index (*R_w_*) [35,36] represents the stability in the process of the combustion, which is delineated as:

(2) $$R_{w}=8.5875\times 10^{7}\times \frac{W_{max}}{T_{s}\times T_{p}}$$

where *W_max_*, *T_s_* and *T_p_* represent the maximum mass loss rate, the starting temperature and the peak temperature, respectively. The combustion stability of mixtures is increasing with the value of *R_w_* [37].

The full experimental scheme is listed as Table 3, and each test is run in duplicate and ±error indicates as the standard deviation of the two results to keep the experiment repeatable and dependable.

## 3. Results and Discussion

### 3.1. TG-DTG Curves and SEM Diagrams of Different Experiments

**Figure 4:**
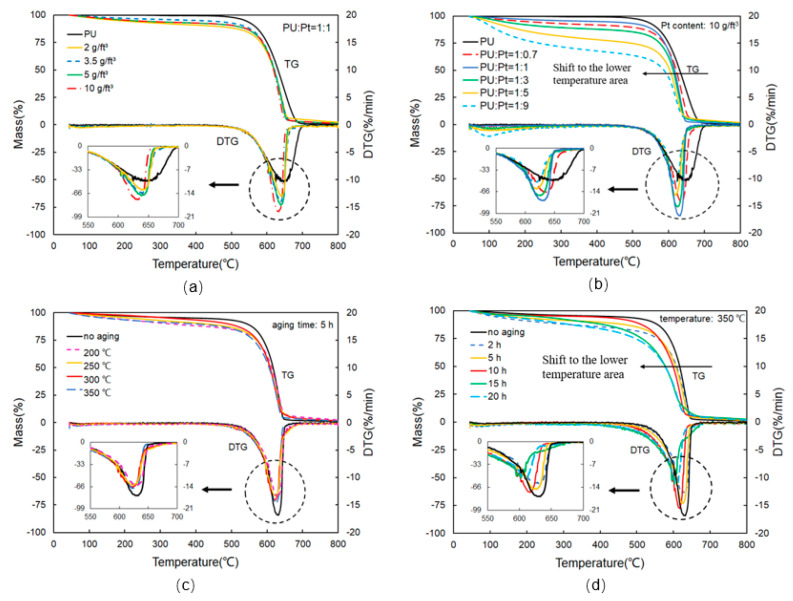
TG-DTG curves in different experiments: (**a**) different Pt contents of PU/catalyst mixtures; (**b**) different ratio of PU/Pt mixtures; (**c**) different thermal aging temperatures; (**d**) different thermal aging times.

The influences of different Pt content and different mass ratios of PU/Pt mixtures are studied by thermogravimetric experiments from Figure 4a,b. There are two main stages divided by the weight loss process of PU after adding Pt/Al_2_O_3_ catalyst. During the first stage ranging from 45 to 500 °C, moisture in the mixtures is evaporated with the increase in heating temperature. The easily oxidized substances such as oxygen-containing functional groups on the surface of PU particles start to become lost. The second stage varies from 500 to 800 °C, in which PU particles undergo a rapid oxidation and the TG curves come down noticeably. The oxidation rate of mixtures is promoted, and the curves shift to the lower temperature area with the increase in Pt content, and the 10 g/ft^3^ PU/Pt mixture has the faster reaction rate. Figure 4b compares the TG–DTG curves at different blending ratios. When the blending ratio of PU/Pt is beyond 1:1, the mass loss and oxidation rate of the PU/Pt mixtures increase more slowly than the mixtures with scaling below 1:1. The rate of oxidation reaches the maximum value when the ratio of PU/Pt is 1:1 because the proportion of Pt/Al_2_O_3_ catalyst for improving the PU particles’ combustion may be saturated.

The influences of different aging temperatures and times on soot combustion are studied from Figure 4c,d. The reaction rate of the aging mixtures is slower than the no-aging PU/Pt mixture, and it increases gradually with the increasing temperature and the maximum value occurs at 350 °C. From Figure 4d, with the aging time increasing, the tendency to shift to the lower temperature area is more apparent. When the aging time is 15 h, the shift amount to the lower temperature area attains the maximum value.

**Figure 5:**
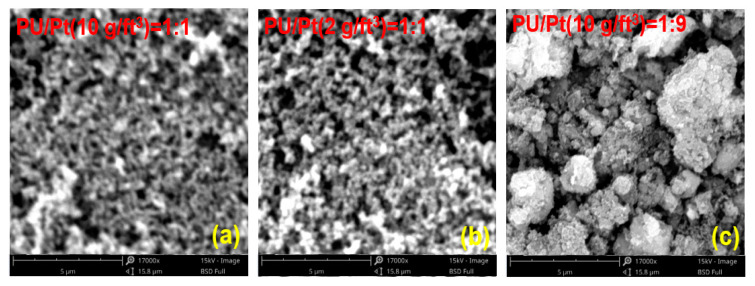
SEM diagrams in no aging experiments: (**a**) PU/Pt (10 g/ft^3^) = 1:1; (**b**) PU/Pt (2 g/ft^3^) = 1:1; (**c**) PU/Pt (10 g/ft^3^) = 1:9.

Figure 5 shows the SEM diagram of no-aging mixtures applied in the thermogravimetric experiments. As shown in the figure, there is no obvious difference between the morphology of PU/Pt mixtures at 2 g/ft^3^ and 10 g/ft^3^. The PU particles and Pt/Al_2_O_3_ catalyst are relatively uniform in size and well-dispersed when the mass ratio is 1:1. When the mass ratio of PU/Pt mixture is 1:9, a high agglomeration between particles is observed from Figure 5c. Therefore, if the Pt content in the PU/Pt mixtures is too much, the large particles will agglomerate, which makes it difficult for the surface of the PU particles to make contact with oxygen molecules. This is similar to the conclusion in Figure 4a,b, which suggests that when the uniformity and dispersion of particles are better, the combustion performance is more sufficient.

**Figure 6:**
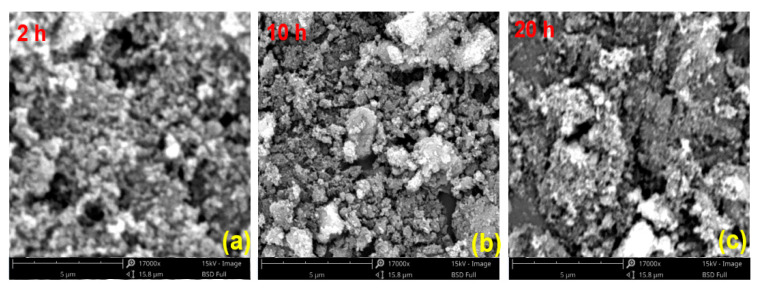
SEM diagrams in different aging times at 350 °C with PU/Pt (10 g/ft^3^) mixtures at 1:1 mass ratio: (**a**) 2 h; (**b**) 10 h; (**c**) 20 h.

Figure 6 shows the SEM diagram of PU/Pt mixtures at different aging times. It can be seen that after aging treatment, the agglomeration phenomenon between particles increases obviously, and the uniformity is greatly damaged. Compared with Figure 6a, when the aging time is 10 h, the agglomeration between the PU and catalyst increases and the particles size distribution is not uniform as a whole, but the well-dispersed phenomenon is still observed in some local ranges. When the aging time is 2 h, the tight-contact area between the particles reduces and the empty area increases. This phenomenon is similar with Figure 5c,d, the agglomeration between PU and Pt particles increases when the PU/Pt mixtures undergo the thermal aging process, which may cause the oxidation rate of aging-samples decrease.

### 3.2. Influence of Different Contents of Pt on Soot Combustion

**Figure 7:**
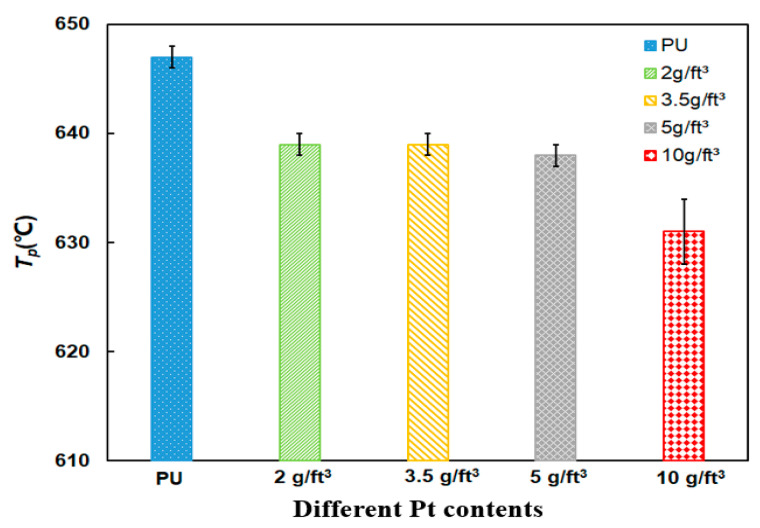
Comparison of *T_p_* of PU/Pt mixtures at different contents of Pt.

The peak temperature is another objective index which represents the maximum weight loss rate. Compared to the *T_p_* of PU particles at 647 °C, the *T_p_* of 10 g/ft^3^ Pt content of PU/Pt mixture is 632 °C from Figure 7, which reduces 15 °C. The main reason can be explained that the larger Pt content mixture is more likely to form the higher vacancy concentrations and facile oxygen desorption, which is beneficial for PU particles to be oxidized earlier and rapidly [36]. The 10 g/ft^3^ Pt content of catalyst are applied in the subsequent experiments.

**Figure 8:**
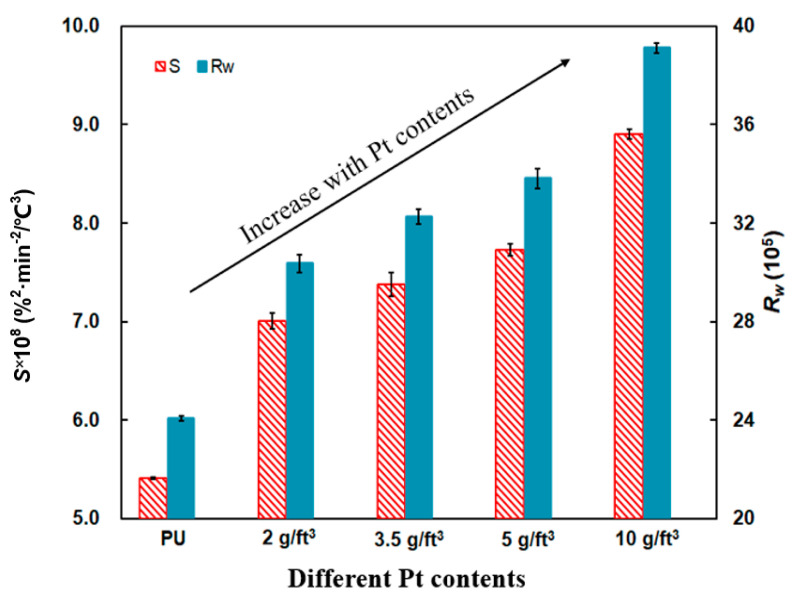
Comparison of the *S* and *R_w_* indices of PU/Pt mixtures at different Pt contents.

Figure 8 makes a distinction among different Pt contents of PU/Pt mixtures with *S* and *R_w_*, and two of the indices increase compared those of PU particles, which suggests Pt/Al_2_O_3_ catalyst is able to promote the oxidation of soot obviously. When the Pt content is 10 g/ft^3^, *S* and *R_w_* reach the maximum values. Compared with the *S* and *R_w_* of PU particles at 5.41 × 10^−8^ %^2^min^−2^°C^−3^ and 24.06 × 10^5^, the *S* and *R_w_* of PU/Pt mixture with 10 g/ft^3^ are 8.90 × 10^−8^ %^2^min^−2^°C^−3^ and 39.11 × 10^5^, which is an improvement of 54.71% and 52.83%, respectively.

This phenomenon is similar with Luo’s experiments [37]. The reason of two indices’ increases is probably related to the physicochemical properties of the Pt/Al_2_O_3_ catalyst [36,38]. Under N_2_/O_2_ atmosphere, a small part of Pt atoms can interact with the anchor sites of five-coordinated Al^3+^ ions on high content of Pt loading [39,40]. With the clustering of Pt particles, the active phase of CO is promoted, and the activity of the Pt catalyst increases due to the increased oxygen concentration [20,37,41]. It is reported from Han et al. [42] that the morphology of soot is strongly dependent on its position and oxygen concentration. Therefore, the ability of promoting the soot oxidation becomes stronger with 10 g/ft^3^ mixture because of its higher oxygen concentration.

### 3.3. Influence of Catalyst Blending Ratio on Soot Combustion

**Figure 9:**
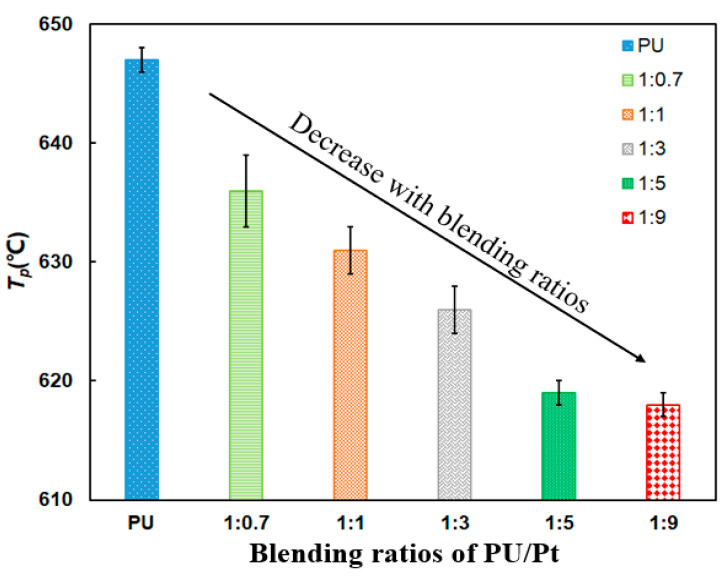
Comparison of *T_p_* of PU/Pt mixtures at different blending ratios.

It is a strange phenomenon from Figure 9 that the *T_p_* decreases gradually with the increase in the Pt catalyst proportion. It attains to the minimum value at 618 °C when the ratio is 1:9, which is reduced by 29 °C compared with pure PU particles. This tendency is probably caused by the strong interaction between Al_2_O_3_ support and Pt particles, which improves the low temperature reducibility of Pt oxides and the catalytic performance of soot oxidation [36]. However, the oxidation rate of 1:9 PU/Pt mixture is the slowest one from Figure 9, because the more Pt catalyst may block the contact of PU particles surface with oxygen. As a result, the *S* and *R_w_* indices decrease comprehensively.

**Figure 10:**
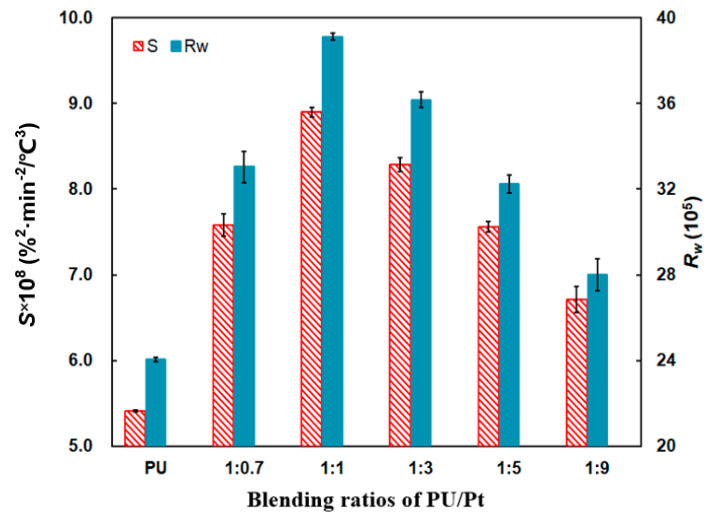
Comparison of the *S* and *R_w_* indices at different blending ratios.

The bar chart in Figure 10 makes an obvious comparison of the *S* and *R_w_* indices. The *S* and *R_w_* indices significantly increase at any ratio compared with PU particles, which indicates that the addition of the Pt catalyst promotes the soot oxidation. The *S* and *R_w_* apparently increase, and the maximum values of *S* and *R_w_* occur at the 1:1 weight ratio at 8.90 × 10^−8^ %^2^min^−2^°C^−3^ and 39.11 × 10^5^, respectively. When the weight ratios are beyond 1:1, the extent of promotion weakens compared to the PU/Pt mixtures with a 1:1 ratio. The tendency of the two indices varying from 1:1 to 1:9 increase firstly after declining, and they reach the maximum values at the weight ratio of 1:1.

This may be explained that the PtO_2_ particles and large Pt microcrystals can be formed and reduced easily with the increase in the amount of Pt catalyst. In the process of reduction, the oxygen atoms adsorbed on the surface can be generated in a quick and easy way [20,36,43]. With those oxygen atoms, the PU/Pt mixtures in proper ratios below 1:1 can carry out more oxygen atoms to improve the oxidation performance. However, when the weight ratios are beyond 1:1, the uniformity of mixtures is not so superior, and too many Pt catalysts may block the contact area between the PU particles and oxygen. The oxygen atoms adsorbed on the surface of the PU particles may finally show a saturated tendency [38,43]. The contact area reduces because of the reduced oxygen adsorption capacity and the blockage of the Pt catalyst [42,44], which negatively affects the increase in the *S* and *R_w_* indices obviously. As a result, the PU/Pt mixture at 1:1 is applied in the next experiments.

### 3.4. Influence of Different Thermal Aging Temperatures on Soot Combustion

**Figure 11:**
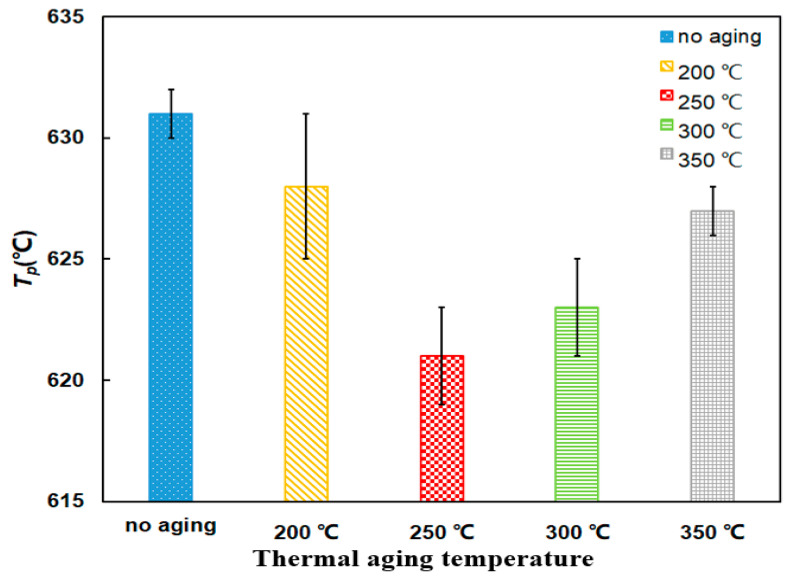
Comparison of *T_p_* of PU/Pt mixtures at different thermal aging temperatures.

Compared with the no aging PU/Pt mixture, the *T_p_* of aging mixtures decrease at first and then increase. It reaches the lowest point at 621 °C when aging temperature is 250 °C from Figure 11, which suggests the PU/Pt mixtures heated for 5 h tend to make PU particles start to oxidize more easily. However, the reaction rate is so slow that the overall combustion performance is not good as the no aging mixture.

**Figure 12:**
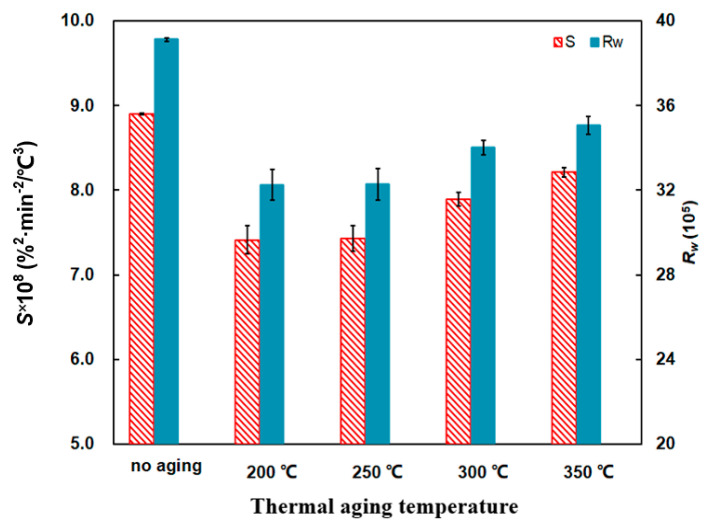
Comparison of the *S* and *R_w_* indices of at different thermal aging temperatures.

From Figure 12, the ensemble trend is that the *S* and *R_w_* indices decrease at first and then increase along with the temperature. The two indices of the PU/Pt mixture heated at 350 °C for 5 h is 8.21 × 10^−8^ %^2^min^−2^°C^−3^ and 35.06 × 10^5^, which is a 7.75% and 10.36% decrease, respectively, compared with the no aging mixture. This indicates that the promoted effect of aging mixtures on soot combustion is weaker than that of no aging, but those aging mixtures still have a positive catalytic role in the oxidation of pure PU particles.

The microstructure of aging PU particles turns to a more orderly form in which the specific adsorption surface may decrease [26,45], and the oxygen atoms absorbed by the mixture’s surface decreases. The aging PU/Pt mixtures have milder catalytic promotion than the no aging mixture on soot combustion with those chemical–physical changes. Those deeper reasons will be studied at the next project, and the aging mixture at 350 °C is applied in next experiments.

### 3.5. Influence of Different Thermal Aging Times on Soot Combustion

**Figure 13:**
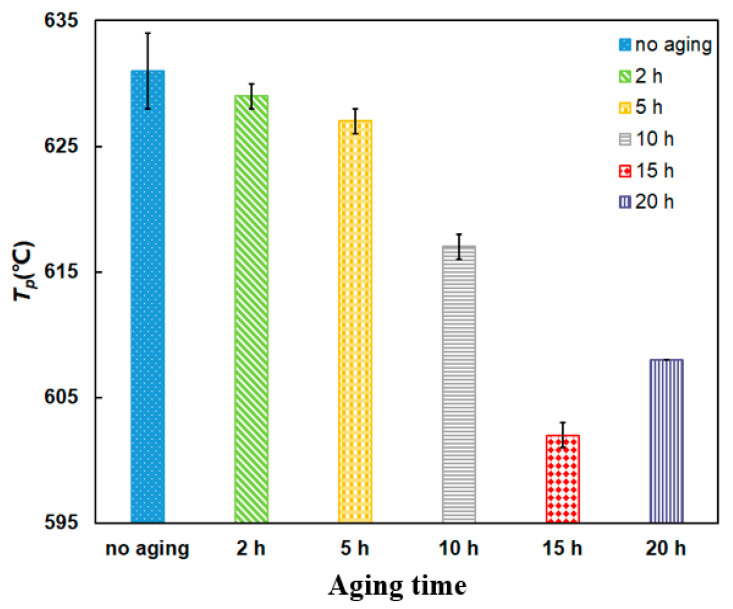
Comparison of *T_p_* of PU/Pt mixtures at different thermal aging times.

A comparison of the *T_p_* is shown in Figure 13. The *T_p_* fluctuates below 635 °C and all the *T_p_* of aging mixtures are lower than that of the no aging mixture, which indicates that the aging mixtures can improve the soot ignition performance. When the aging time is 15 h, the value of *T_p_* reaches the minimum point at 602 °C, which reduces by 29 °C. This may be explained that the Pt catalyst after thermal aging process has the better low-temperature area reduction performance than the no aging mixture [36,43]. This indicates the Pt atoms can be oxidized to different forms of Pt oxides, such as PtO, PtO_2_ and PtO_2_ [45], during the thermal aging process. The Pt oxides’ reduction can release the oxygen atoms, which decreases the *T_p_* of PU particles during the regeneration process.

**Figure 14:**
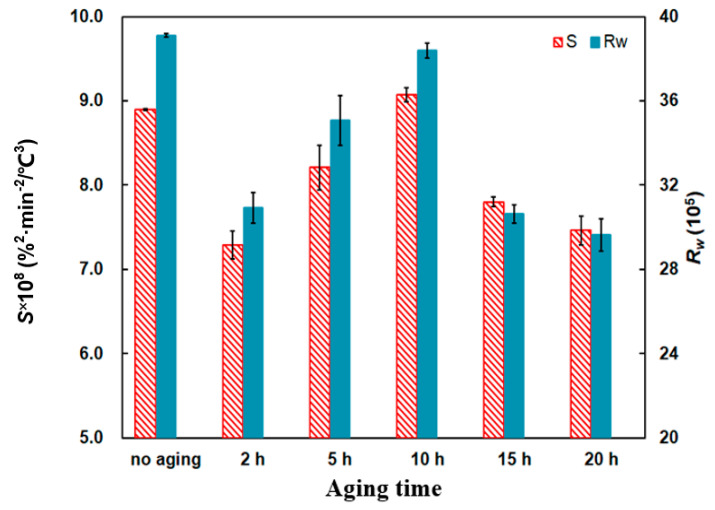
Comparison of the *S* and *R_w_* indices at different thermal aging times.

From Figure 14, the *S* and *R_w_* indices show the comparisons. Compared to the no-aging mixture, two indices of aging mixtures decrease to a greater or lesser degree. When the aging time is 10 h, the *S* and *R_w_* are 9.07 × 10^−8^ %^2^min^−2^°C^−3^ and 38.39 × 10^5^, respectively. This indicates that the combustion performance promotes to a modest extent but the combustion stability of PU/Pt mixtures decreases.

The catalytic activity of soot oxidation is affected by two factors, including the contact efficiency between the catalyst active sites and soot as well as the density and properties of the active sites [39]. When the PU/Pt mixtures are heated, the catalyst active sites may reduce slightly [46] so that the *S* and *R_w_* indices decrease. In the previous studies, when the PU particles are appropriately thermal heated, the PU particles have a stronger ability to infiltrate into the Pt catalyst if the staying duration is long enough [37,47]. The combustion performance of 10 h aging the PU/Pt mixture at 350 °C bears a resemblance to the no-aging PU/Pt mixture.

## 4. Conclusions

The soot combustion characteristics in the presence of a Pt/Al_2_O_3_ catalyst after the thermal aging process are investigated by the TGA and fixed-bed apparatus. The following main results are drawn based on this preliminary work:

(1) Compared with the other different Pt contents (2 g/ft^3^, 3.5 g/ft^3^ and 5 g/ft^3^) in the PU/Pt mixtures tests, the ability of promoting the soot oxidation becomes stronger with 10 g/ft^3^ Pt content mixture, because 10 g/ft^3^ PU/Pt mixture can absorb more oxygen atoms.
(2) When the PU/Pt weight ratio is 1:1, the Pt/Al_2_O_3_ catalyst has the greatest effect on improving soot combustion. The comprehensive combustion index *S* and combustion stability index *R_w_* increase to 8.90 × 10^−8^ %^2^min^−2^°C^−3^ and 39.11 × 10^5^, respectively.
(3) With the increase in aging temperature from 200 to 350 ℃ for 5 h, the promoting of soot oxidation increases, compared to pure PU particles. When the aging temperature is 350 ℃, the *S* and *R_w_* indices reach the maximum values at 8.21 × 10^−8^ %^2^min^−2^°C^−3^ and 35.06 × 10^5^, respectively. Even though the improving effect of those aging mixtures are not as good as the no aging PU/Pt mixture.
(4) When the aging time is from 2 to 20 h at 350 °C, the tendency of shifting to the lower temperature area is more obvious. When the aging time is 10 h, the *S* and *R_w_* indices reach the maximum values at 9.07 × 10^−8^ %^2^min^−2^°C^−3^ and 38.39 × 10^5^, respectively.

The practical significance of this study is to explore that the catalytic activity and thermal aging effect on soot and the soot/catalyst mixtures in an O_2_/N_2_ atmosphere by means of TGA and the fixed-bed apparatus. The results indicate that compared with the no aging mixture, the soot combustion promotion of the PU/Pt mixtures after thermal aging process decreases. There is no doubt that a lot of basic research work needs to be done in the unexplored aspects of soot combustion. According to the practical PM deposited in the DPF, the effect of thermal aging on soot combustion process with catalyst is worthy of future investigation. To investigate the chemical–physical properties of the samples after the thermal aging process, there are some deep mechanisms that need to be explored in the next project.

## Figures and Tables

**Table 1:** Detailed parameters of PU.

Soot	Average Diameter (nm)	BET (m^2^/g)	Oil Absorption (g/100 g)	Ash Content (%)
PU	25	92	460	0.02

**Table 2:** Main parameters of catalysts.

Serial Number	Composition	Pt Contents	Average Diameter (nm)
1#	Pt/Al_2_O_3_	2 g/ft^3^	50 ± 10
2#	Pt/Al_2_O_3_	3.5 g/ft^3^	50 ± 10
3#	Pt/Al_2_O_3_	5 g/ft^3^	50 ± 10
4#	Pt/Al_2_O_3_	10 g/ft^3^	50 ± 10

**Table 3:** The summary of characteristic parameters of all samples in this study.

Case #	PU/Catalyst	wt. Ratio	Aging Temperature (°C)	Aging Time (h)	*T*_s_ (°C)	*T*_e_ (°C)	*T*_p_ (°C)	*W*_max_ (%/min)	*S* × 10^8^ (%^2^min^−2^°C^−3^)	*R_w_* (10^5^)
1–2	PU:—	—	—	—	573 ± 1	684 ± 1	647 ± 1	10.29 ± 0.11	5.39 ± 0.02	24.06 ± 0.19
3–4	PU:Pt (2 g/ft^3^)	1:1	—	—	575 ± 1	656 ± 2	639 ± 1	12.99 ± 0.12	7.01 ± 0.27	30.36 ± 0.31
5–6	PU:Pt (3.5 g/ft^3^)	1:1	—	—	581 ± 2	655 ± 1	639 ± 1	13.95 ± 0.09	7.38 ± 0.12	32.26 ± 0.15
7–8	PU:Pt (5 g/ft^3^)	1:1	—	—	582 ± 0	653 ± 1	638 ± 1	14.62 ± 0.25	7.73 ± 0.17	33.81 ± 0.26
9–10	PU:Pt (10 g/ft^3^)	1:1	—	—	578 ± 1	651 ± 2	631 ± 4	16.84 ± 0.52	8.90 ± 0.26	39.11 ± 1.17
11–12	PU:Pt (10 g/ft^3^)	1:0.7	—	—	578 ± 2	653 ± 2	636 ± 5	14.14 ± 0.32	7.58 ± 0.16	33.03 ± 0.71
13–14	PU:Pt (10 g/ft^3^)	1:3	—	—	576 ± 2	646 ± 1	626 ± 2	15.19 ± 0.14	8.29 ± 0.08	36.17 ± 0.36
15–16	PU:Pt (10 g/ft^3^)	1:5	—	—	561 ± 5	641 ± 0	619 ± 1	13.04 ± 0.26	7.56 ± 0.06	32.23 ± 0.43
17–18	PU:Pt (10 g/ft^3^)	1:9	—	—	548 ± 1	641 ± 1	618 ± 1	11.04 ± 0.27	6.71 ± 0.17	27.99 ± 0.75
19–20	PU:Pt (10 g/ft^3^)	1:1	200	5	574 ± 1	649 ± 2	628 ± 1	13.54 ± 0.14	7.41 ± 0.012	32.28 ± 0.17
21–22	PU:Pt (10 g/ft^3^)	1:1	250	5	573 ± 0	642 ± 1	621 ± 2	13.38 ± 0.24	7.43 ± 0.07	32.29 ± 0.19
23–24	PU:Pt (10 g/ft^3^)	1:1	300	5	571 ± 3	641 ± 1	623 ± 1	14.09 ± 0.29	7.89 ± 0.13	34.01 ± 0.28
25–26	PU:Pt (10 g/ft^3^)	1:1	350	5	570 ± 2	640 ± 2	627 ± 1	14.59 ± 0.39	8.21 ± 0.27	35.06 ± 0.58
27–28	PU:Pt (10 g/ft^3^)	1:1	350	2	566 ± 2	642 ± 1	629 ± 1	12.82 ± 0.26	7.29 ± 0.14	30.92 ± 0.26
29–30	PU:Pt (10 g/ft^3^)	1:1	350	10	564 ± 1	631 ± 0	617 ± 1	15.56 ± 0.08	9.07 ± 0.013	38.39 ± 0.012
31–32	PU:Pt (10 g/ft^3^)	1:1	350	15	519 ± 2	620 ± 3	602 ± 1	11.14 ± 0.19	7.80 ± 0.029	30.61 ± 0.15
33–34	PU:Pt (10 g/ft^3^)	1:1	350	20	528 ± 2	622 ± 1	608 ± 0	11.06 ± 0.27	8.68 ± 0.037	29.63 ± 0.21

## Data Availability

The data presented in this study are available on request from the corresponding author.
